# Comparison of shoe-length fit between people with and without diabetic peripheral neuropathy: a case–control study

**DOI:** 10.1186/1757-1146-5-9

**Published:** 2012-04-16

**Authors:** Alistair D McInnes, Farina Hashmi, Lisa J Farndon, Amanda Church, Maria Haley, Debora M Sanger, Wesley Vernon

**Affiliations:** 1 Division of Podiatry, School of Health Professions, University of Brighton, Brighton, UK; 2 Primary and Community Care Services, Sheffield Teaching Hospitals NHS Foundation Trust

## Abstract

**Background:**

Amongst the many identified mechanisms leading to diabetic foot ulceration, ill-fitting footwear is one. There is anecdotal evidence that people with diabetic peripheral neuropathy wear shoes that are too small in order to increase the sensation of fit. The aim of this study was to determine whether people with diabetic sensory neuropathy wear appropriate length footwear.

**Methods:**

A case–control design was used to compare internal shoe length and foot length differences between a group of people with diabetes and peripheral sensory neuropathy and a group of people without diabetes and no peripheral sensory neuropathy. Shoe and foot length measurements were taken using a calibrated Internal Shoe Size Gauge® and a Brannock Device®, respectively.

**Results:**

Data was collected from 85 participants with diabetes and 118 participants without diabetes. The mean difference between shoe and foot length was not significantly different between the two groups. However, a significant number of participants within both groups had a shoe to foot length difference that lay outside a previously suggested 10 to 15 mm range. From the diabetic and non-diabetic groups 82% (70/85) and 66% (78/118), respectively had a foot to shoe length difference outside this same range.

**Conclusions:**

This study shows that although there is no significant difference in shoe-length fit between participants with and without neuropathy, a significant proportion of these populations wear shoes that are either too long or too short for their foot length according to the 10 to 15 mm value used for comparison. The study has highlighted the need for standardised approaches when considering the allowance required between foot and internal shoe length and for the measurement and comparison of foot and shoe dimensions.

## Background

Diabetic foot ulceration is associated with increased morbidity and higher mortality rates [1-3]. Jeffcoate and Harding [4] and Pecoraro et al [5] found that in more than 80% of cases, amputation was preceded by foot ulceration. The financial burden is considerable and Gordois et al [6] identified that foot ulcerations and amputations cost the UK National Health Service (NHS) £244 million in 2001.

Boulton [7] has suggested that the lifetime risk of those with diabetes developing a foot ulcer is as high as 15%. There is now substantial evidence that diabetic neuropathy is a major aetiological factor for diabetic foot ulceration [8]. The mechanisms leading to foot ulceration are either extrinsic (e.g. undetected trauma) or intrinsic to the foot (e.g. neuropathy contributing to foot deformity resulting in high multidirectional pressures during gait) [9,10]. Moulik et al [11] reported the presence of neuropathy in 61% of patients presenting to a foot clinic for the first time with a foot ulcer.

Other identified factors that contribute to foot ulcer risk include body weight and footwear [12]. Footwear has been shown to be a major contributing factor not only in the development of foot ulceration but also subsequent amputation [13-16]. One descriptive study reported that approximately half of the participants with diabetes and peripheral sensory neuropathy had a footwear-related event that led to limb amputation [17].

There is much anecdotal evidence that people with diabetic neuropathy often wear shoes that are too small in order to increase the sensation of fit [18]. Litzelman demonstrated that diabetic patients with insensate feet tend to buy and wear overly tight shoes, and at least 25% of people with Type 2 diabetes wear inappropriately sized footwear [15]. Published footwear studies of participants with diabetes use variable criteria to measure both the foot and the shoe and also to describe inappropriately fitting footwear [15,19-22]. There is no unified opinion regarding what the appropriate ‘gap’ between the distal point of the foot and the shoe should be in order for it to be considered an appropriate fit. This ‘gap’ seems to be arbitrary in length and dependent on various shoe manufacturers’ sizing systems. The work conducted by Chantelau and Gede [21] provides an example of this gap being 10 to 15 mm in order to allow "extra space for the toes when extending during walking and standing". While a different value has been suggested by DiMaggio and Vernon [23], their experience-based suggestion came after completion of the data collection phase of this study.

The aim of this study was to determine whether people with diabetic sensory neuropathy wear appropriate length footwear compared with a control group. In this study Chanteleau and Gede's example value of 10 to 15 mm for the difference between foot and shoe length was used as the standard. Although Chantelau and Gede’s suggestion was not in itself evidence-based, it has been previously used and quoted by others, hence its use in this study. The hypothesis tested was: participants with diabetic peripheral neuropathy wear shorter footwear in relation to foot length compared to those without diabetes and peripheral neuropathy. This study was intended to build on the suggestions of others and provide more useful evidence for those involved in the care of the diabetic foot.

## Methods

### Study design

A case–control design was implemented to compare the shoe-length fitting differences between two groups: (i) individuals who had both diabetes and peripheral sensory neuropathy, and (ii) individuals who did not have diabetes or peripheral sensory neuropathy. Ethical committee and research governance approval was sought and granted before the study commenced. All participants gave written informed consent before entering the study. The investigators responsible for recruitment and data collection were provided with common training regarding the measurement equipment used. Ethical approval was given by North Staffordshire Research and Ethics Committee (Reference 06/Q2604/162).

### Study sample and recruitment

There is little published data concerning the relationship between foot and shoe size in people with diabetes and peripheral neuropathy. Harrison et al [22] considered foot length amongst other factors, however the measurements were all taken with participants in a standing position and major differences were found in relation to width measurements. In addition, it is not clear what, if any, allowances were made by the authors in comparing foot versus shoe length. It is also possible that the authors relied solely on the manufacturers (variable) stated length for footwear. Because of the lack of data in this area, a sample size calculation was not performed to ascertain the minimum number of participants required to detect clinically meaningful findings using statistical analysis.

In the absence of a sample size calculation, recruitment figures were initially estimated using existing audit data from one of the research sites (the Leaf Hospital, Eastbourne, UK). The response rate for research participation by the patients at this hospital is known to be 40 to 60%. Therefore, it was anticipated that 400 patients with diabetes would be invited to take part, which would translate to 160 and 240 people responding (according to the figures quoted above). Prior to the study commencing, it was envisaged that a similar number of participants for the comparison group would also be recruited. However, under recruitment was experienced due to time restrictions.

Patients with diabetes who were willing to participate in the study were recruited from the podiatry service at the Sheffield Northern General Hospital. Approximately 70% of patients attending this service were ineligible for participation as they were wearing footwear prescribed by the hospital orthotist (see exclusion criteria below). The non-diabetic participants were recruited from clinics at the Leaf Hospital, Eastbourne, UK. The same recruitment procedures were employed at both study sites where strict adherence to protocol was maintained.

Participants were eligible if they had Types 1 or 2 diabetes (with peripheral sensory neuropathy) or were healthy and did not have diabetes (with no sensory neuropathy) and between the ages of 40 and 75 years. Both males and females were included in the study. Exclusion criteria were: history of foot surgery; use of bespoke footwear; use of more than three different pairs of shoes in one week; use of dressings that may interfere with the measurement of the foot (e.g. an ulcer dressing on the first toe); rheumatoid arthritis, stroke, neurological disorders, musculoskeletal disease or major systemic arthropathy.

All participants were asked to arrive to the clinic wearing the shoes that they most commonly wore outdoors.

### Assessment of peripheral neuropathy

All participants were assessed for the presence or absence of peripheral sensory neuropathy of both feet by evaluating symptoms and measuring specific clinical signs using the neuropathic symptom score [24,25], the neuropathic disability score [26] and vibration perception threshold values [27].

The neuropathy symptom score (NSS) was used to assess the symptoms of neuropathy. An NSS score of greater than or equal to 3 was considered abnormal (i.e. confirmation of sensory neuropathy). The neuropathy disability score (NDS) was used to assess for signs of neuropathy. A score of 6 or above on the NDS [28] was considered abnormal. Vibration perception was evaluated using a 128 Hz tuning fork at three points (plantar aspect of the hallux, 1^st^ and 5^th^ metatarsal heads) on each foot. A 10 gram monofilament was used to test for pressure sensation at four points on each foot (hallux, 1^st^, 2^nd^ and 5^th^ metatarsal heads). An absence of the detection of pressure at one site or more indicated sensory neuropathy. A positive outcome for each of these tests indicated peripheral neuropathy and therefore, inclusion or exclusion to the study depending on whether or not the person had diabetes.

### Outcome measures

The primary outcome measure was that of the difference between the foot and shoe length for both groups. The secondary outcome measure was the proportion of participants whose foot to shoe length difference was outside the 10 to 15 mm range.

### Foot measurements

**Figure 1:**
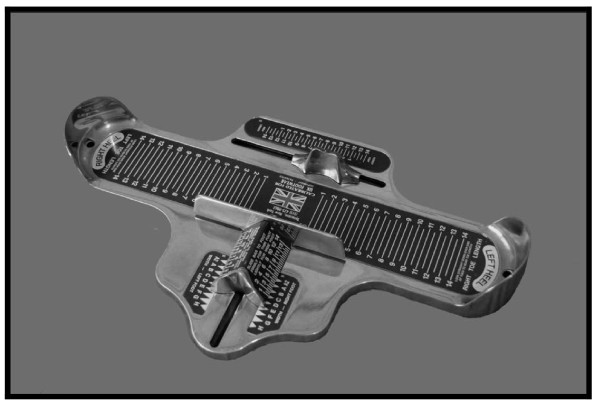
**The Brannock® device.**

Both feet of each participant were measured using a Brannock Device® (Algeos, UK). Each participant stood barefoot and relaxed, with the feet slightly apart and with the weight evenly distributed between both feet. Using the Brannock Device® (Figure 1) the foot being measured was lifted and placed onto the base with the heel being firmly located against the back of the heel cup of the device with the researcher firmly holding the subject’s ankle and heel cup together. The researcher then pressed the subject's toes flat against the scale of the device, looking vertically down on the longest toe (at right angles) to note the foot length indicated. It is important to note that the longest toe was not necessarily the first toe. Using a fine, non-permanent wipe-clean marker pen and straight-edge, this position was then marked on the device and the distance from the heel of the device to this marked point was measured in millimetres (mm) using a calibrated ruler. The same procedure was repeated for the other foot.

### Footwear measurements

**Figure 2:**
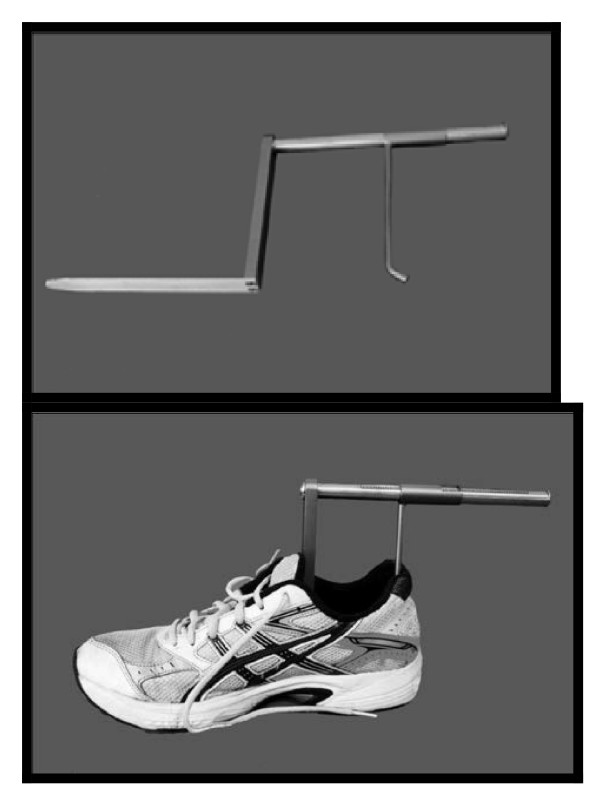
The Internal Shoe Size Gauge.

The subject’s footwear was placed on a firm level surface. A calibrated Internal Shoe Size Gauge® (SATRA, UK) was then placed into the shoe and the flat bar of the device pushed into the shoe until it clearly contacted the end of the toe box (Figure 2). The slide of the device was then adjusted until the rear curved bar section touched the heel of the shoe. The internal length of the shoe was recorded in mm. The same procedure was then repeated for the other shoe. The measure used to test the hypothesis was the difference between the overall foot length and internal shoe-length.

### Statistical analysis

The foot measurement was subtracted from the internal shoe length measurement and the difference recorded in mm. To test whether continuous data were normally distributed residual plots and the Shapiro–Wilk test were used. Data from the diabetes group were not normally distributed, so non-parametric tests were used to confirm significant differences between the two participant groups. For categorical variables the Chi-square test was used and for continuous variables that were not normally distributed the Mann–Whitney U test was used. It was decided a priori to use the 5% two-sided significance level to decide whether to reject the null hypothesis (p < 0.05). Statistical analyses were carried out using SPSS 16.

## Results

**Figure 3:**
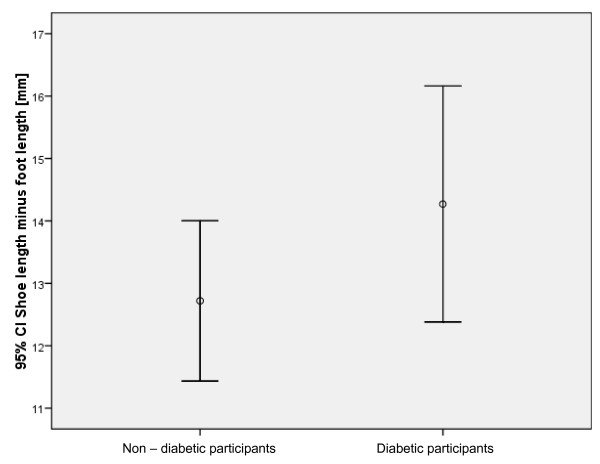
Comparison of the two groups and the difference in the foot and shoe lengths (p = 0.25).

Outcome measurements were collected for 118 control participants (i.e. without diabetes) and 85 people with diabetes and peripheral neuropathy. A summary of descriptive data is provided in Table 1. The difference between internal shoe and foot size was not significantly different between the diabetes and control groups (p = 0.253) (Figure 3). The foot length was significantly greater in the group with diabetes compared to participants without diabetes (p = 0.001), as was the comparison between shoe-lengths (p = 0.001).

**Table 1:** Shoe and foot length data for the diabetic and non-diabetic groups

	**Shoe length [mm]**		**Foot length [mm]**		**Shoe length minus foot length [mm]**	
	**Non – diabetic** **(n = 118)**	**Diabetic** **(n = 85)**	**Non – diabetic** **(n = 118)**	**Diabetic** **(n = 85)**	**Non – diabetic** **(n = 118)**	**Diabetic** **(n = 85)**
Median	277	262	268	249	14	12
Maximum	302	320	290	312	34	37
Minimum	232	243	214	230	−5	0
Interquartile range	21	26	30	25	14	9

**Figure 4:**
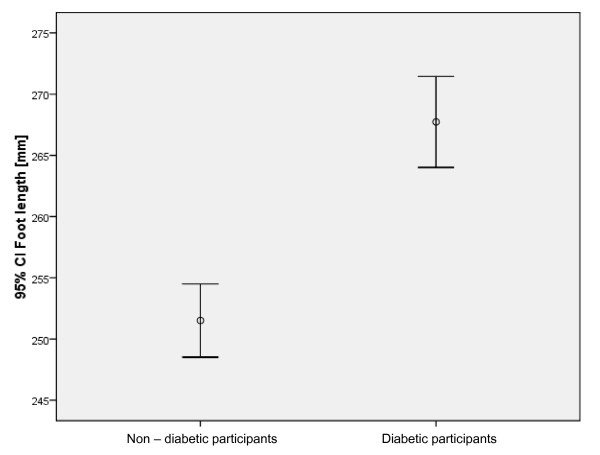
Comparison of the two groups and the difference in the foot lengths (p = 0.001).

**Figure 5:**
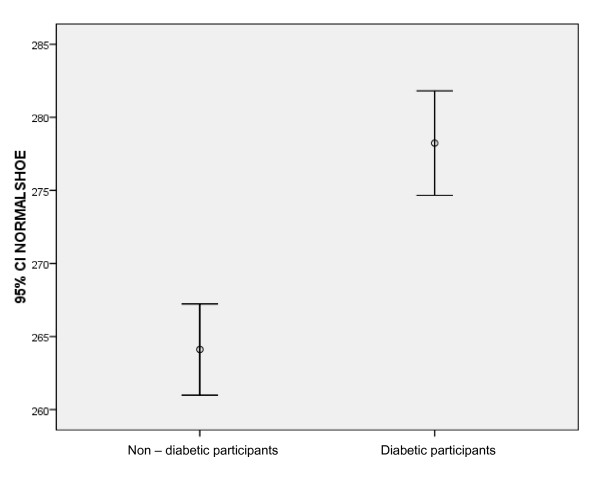
Comparison of the two groups and the difference in the shoe lengths (p = 0.001).

Seventy-eight of the 118 participants without diabetes (66%) had a difference in foot and shoe length outside the 10 to 15 mm range. Of those outside this range, 42 of the 78 participants (55%) had a difference in foot to shoe length that was below 10 mm (range: -5 to 9 mm) and 36 (47% had a difference in foot to shoe length above 15 mm (range: 16 to 34 mm). There was a statistically significant difference in the number of participants in each group that were within and outside the 10 to 15 mm range (p = 0.009) (Figures 4 and 5).

Seventy of the 85 participants with diabetes (82%) had a difference in foot and shoe length outside the 10 to 15 mm range. Fifteen (18%) of these participants had a difference in foot to shoe length within the 10 to 15 mm range. Of those outside this range, 30 of the 70 participants (43%) had a difference in foot to shoe length that was below 10 mm (range: 0 to 9 mm) and 40 (57%) had a difference in foot to shoe length above 15 mm (range: 16 to 37 mm). There was no statistically significant difference between the groups above and below the 10 and 15 mm range (p = 0.182).

## Discussion

This study did not find a significant difference between the foot and internal shoe length in diabetic and non-diabetic subjects. A significant number of participants did, however, have a gap between the toe and shoe that lay outside Chantelau and Gede's [21] suggested 10 to 15 mm range (p = 0.009). Therefore, according to Chantelau and Gede, the majority of the participants (72%) in our study were wearing footwear of incorrect length. In particular, 82% of participants with diabetes were wearing shoes of incorrect length and 35% and 36% of the diabetic and the non-diabetic populations respectively wore shoes that were too short. In our study, 47% of the diabetic population and 31% of the non-diabetic population wore shoes that were too long.

Given the fitting mismatches apparent between foot and internal shoe-length, it is possible that participants may have developed footwear purchasing habits before being diagnosed with diabetes. For example, it is possible that people had their feet measured once as an adult and then continue to purchase the same size shoe over many years without being re-measured, whether or not they have been diagnosed with diabetes. It is also possible that people do not have their feet measured at all, basing their purchases on subjective feelings of fit adequacy alone; a habit that would establish the perceived fit required, and which could continue after receiving a diagnosis of diabetes.

The foot and shoe length differences were significantly greater in terms of overall length in the diabetic group compared to the non-diabetic group. This could relate to a finding by Burns et al [20] who reported that some people purchase a larger shoe size than their foot in order to achieve a wider width fitting, with this practice resulting in a commensurate increase in length. In Chantelau and Gede's study [21], the foot to shoe length matched well with the (continental) shoe size system used, whilst the width sizes did not, which may be another characteristic of an ill-fitting shoe.

Width measurements from a shoe fitting perspective should, however, involve a three-dimensional assessment. Width measurement with a calliper or calliper-like device alone, such as undertaken by Chantelau and Gede [21] and Harrison et al, [22], does not take into consideration the depth needed to accommodate the foot – a consideration that is closely related to the width. As such, shoe width fitting involves a balance between the actual shoe width, foot width, foot depth and available shoe depth and this complexity makes the width dimension more difficult to measure. Therefore, width fitting may be an important factor in correct shoe fitting, but to achieve an acceptable width fitting, the length fitting may need to be compromised.

The gap required between foot and overall internal shoe length of 10 to 15 mm as suggested by Chantelau and Gede is greater than others quoted in the literature (e.g. the 8.5 mm quoted by DiMaggio and Vernon [23]). Both values are intended to allow the foot to extend whilst standing and walking, however these values have also been stated within different fitting contexts. Continental shoe sizing systems are used across mainland Europe, where Chantelau and Gede are based, while US and UK shoe size systems apply to the working context of DiMaggio (US) and Vernon (UK). The US and UK systems half size availability of 4.25 mm allows a greater range of fitting choice between sizes than the Continental system, which allows 2/3 cm (6.66 mm) between size options [29]. As such, with fewer size options in the Continental system, there may be no option other than to recommend a wider allowance from the distal end of the toe to the shoe in order to allow adequate extension of the foot within the shoe. This may explain the reason for Chanteleau and Gede's larger value.

Alternately, it is possible that Chantelau and Gede's value is erroneous. They refer to their 10**–**15 mm value as an example of the gap between foot and shoe-length. However, the data tables presented by Chantelau and Gede appear to indicate gaps predominantly in the order of 6***–***11 mm, with a modal value of 9 mm – a lower range than his suggested example and more in line with the 8.5 mm allowance.

Whichever reason accounts for the differences between Chanteleau and Gede's value and those suggested by others, there is a need for an appropriate standardised measurement and sizing system. Such a system should consider all relevant factors to ensure that a baseline of agreement exists in relation to length and width measurements in the context of foot health.

One inconsistency in the literature is in the measurement of foot to internal shoe-length. The current study used a calibrated internal shoe size gauge (mm)® (SATRA, UK), whilst others have used the following disparate methods, a motorised measuring apparatus [21]; a calibrated (in centimetres) measuring stick and conversion to shoe size [22]; nurse-clinician thumb size to determine end of foot to shoe gap [15]; internal calliper measurements and shoe size [20]; and the use of English shoe size to calibrated measuring stick [19].

In a previous descriptive study of one hundred participants with diabetes, the foot length was measured with the use of a commercial shoe company’s measuring device [22]. The foot length was measured both seated and standing, and the shoe length was measured in centimetres with the use of a calibrated stick, with differences between shoe and foot length being calculated and recorded. The authors concluded that about 33% of patients were more than a half size out in shoe length when seated. One particular finding was that there was no relationship between footwear size and the presence of sensory neuropathy [22]. It is not clear from this article what allowances were made in the study for movements such as slippage when comparing measurements of overall foot and internal shoe-lengths.

The criteria for ‘ill-fitting’ footwear and the methods for measuring the foot and shoe differ widely. This compromises the determination of the prevalence of ill-fitting footwear in a diabetic population as considered in this study. Our study has attempted to determine the relationship of the foot length to the shoe length by taking a standardised approach to both foot and shoe measurements and this approach has highlighted the discrepancies and lack of standardisation in both the measurement techniques and allowances made in shoe fitting.

It is important to note that loose fitting shoes can also potentially cause those pathologies that are related to increased levels of dynamic friction forces between the foot and shoe (e.g. blisters and callus formation), which in the diabetic population can have more serious sequelae. There is sufficient compelling evidence that footwear contributes to foot ulceration [14]. One study [15], investigated the role of footwear in the prevention of foot lesions in people with type 2 diabetes and reached a controversial conclusion that there was no interaction between neuropathy and footwear in the aetiology of foot wounds. However, this was challenged by Chantelau and Gede [21] who suggested that a significant number of people with diabetic neuropathy developed shoe induced foot ulcers.

Despite the studies in this area using different methodologies and taking place within different operating contexts, they have reached similar conclusions; a significant number of people with and without diabetes do not wear appropriately sized shoes. However, the objective identification of specific characteristics of a good fitting shoe remains elusive. A trained shoe fitter can identify characteristics of a shoe that indicate poor fit, but this is very much a craft skill, much of which is not currently supported by research evidence.

This study needs to be viewed in light of a few limitations. Firstly, participant characteristic data, such as height, age and weight, were not recorded, so we were unable to compare the two groups for differences in these variables. There is the possibility that some of these variables (e.g. height) may have had an effect on foot length. If the example of height is explored further, then the significant difference we found in foot length may have simply been due to a difference in height between the two groups. We were, however, primarily interested in the difference between foot length and shoe length, not foot length alone. Secondly, a power calculation (i.e. a sample size calculation) was not performed prior to the study commencing recruitment and the sample size estimate we did make was not reached. This may have affected whether we had sufficient statistical power to detect clinically worthwhile differences between shoe length and foot length. Finally, it was not anticipated that the majority of participants with diabetes and neuropathy would already have bespoke footwear, which resulted in lower numbers of eligible patients being able to enter the study. Further research in this area should consider a longer recruitment time and include more than one centre to ensure that enough participants are able to take part.

## Conclusion

In our study we did not find a significant difference between the foot to internal shoe length relationship of a diabetic and non-diabetic group of adult participants. However, from a clinical perspective, those patients who may be considered at risk of diabetic foot complications can be considered to be wearing footwear that is frequently too short or too long. Further research is required to establish valid criteria for good-fitting footwear; standardise the approach to measure foot and shoe parameters of length, depth and width, and standardise the required gap between the foot and shoe length in order to determine ideal shoe-length. It is possible that certain characteristics of footwear design may interact with the vulnerable foot (i.e. one that has neuropathy) as opposed to characteristics that are inherently ‘ill-fitting’. In addition to this, further work to test and validate the craft knowledge of the shoe fitter is pertinent. Whilst the role of the effects of therapeutic footwear has been evaluated [30] and the effect of footwear as a contributing factor in the pathway to diabetic foot ulceration has been identified [14], the lack of standardisation of footwear measurement prevents recording of the true prevalence of ill-fitting footwear in the diabetic population.

## Declaration of competing interests

The authors declare that they have no competing interests.

## Authors’ contributions

AC participated in the recruitment of volunteers for the study and data collection. LF participated in the co-ordination of the study; interpretation of data and revising the manuscript. FH participated in the design and co-ordination of the study, performed the statistical analysis, and contributed to drafting the manuscript. AM conceived and participated in the design of the study. WV conceived and participated in the design of the study. DS participated in the recruitment of participants for the study and data collection. MH participated in the recruitment of participants for the study and data collection. All authors read and approved the final manuscript.
